# Supplementary material for: A Comparison between the Molecularly Imprinted and Non-Molecularly Imprinted Cyclodextrin-Based Nanosponges for the Transdermal Delivery of Melatonin
Source: Polymers (Basel). 2023 Mar 20;15(6):1543. doi: 10.3390/polym15061543 (PMC10057034; doi:10.3390/polym15061543)
Supplement: Supplementary file 1 [file polymers-15-01543-s001.zip › polymers-2233038-supplementary-correction.pdf]

Supplementary Material

S1. Computational details

All of the simulations were carried out with xTB code (extended Tight Binding) v. 6.4.0 at a semi-empirical level of theory xTB-GFN2 [65,66]. The lowest energy structures were obtained by a conformational search employing the submodule CREST [67] (Conformer-Rotamer Ensemble Sampling Tool), as already applied in our previous study [54].
In detail, the conformational search was run on melatonin, β-cyclodextrin, and the β-cyclodextrin/melatonin inclusion complex, with the default settings to find the lowest energetic minimum for each molecule. During the conformational search on the inclusion complex, only a limited number of atoms was left free to move because of the flexibility of the considered systems. Constraints were added to the atoms composing the pyranose ring of the glucose monomer units, corresponding to the carbons named C_1, C_2, C_3, C_4, C_5, the O atom linking C_1/C_5, and the O atom involved in the 1,4-glycosidic bond. For the inclusion complex, the NCI (*Non-Covalent Interaction*) mode was employed. This option had reduced parameters, specifically for the description of complexes with non-covalent interactions. NCI performed a lesser value of metadynamics (with an increased total time) to the CREST normal mode and included an optimized version of the bias potential for non-covalent complexes. Structures obtained by CREST in the range of 6 kcal/mol (default setting) were then re-optimized in the gas phase at a GFN2 level using tighter thresholds: energy convergence $E_{conv} = 1 \cdot 10^{-7}$ Hartree·Bohr^{-1}, gradient convergence $G_{conv} = 2 \cdot 10^{-4}$, Hartree·Bohr^{-1}, and accuracy for integral cutoffs = 0.01. Inclusion complexes were formed by the manual insertion of guest melatonin into the cavity and with the automatic energy exploration performed by CREST. Vibrational frequencies were numerically computed to ensure that structures were minimal on the Potential Energy Surface (i.e. all real frequencies) and to simulate the IR spectrum of the lowest-energy inclusion complex.

S2. Synthesis of Cyclodextrin-Based Molecularly Imprinted and Non-Molecularly Imprinted Nanosponges (MIP-NSs and NIP-NSs)

Citrate-cyclodextrin-based nanosponges (CD-based NSs) were synthesized by the five-membered cyclic anhydride intermediate formation. The cyclic anhydride was formed, when the citric acid was dehydrated during the heating process, and lost a water molecule, as the first step in the cross-linking of cyclodextrins (CDs) with citric acid (Figure S1 a)). Another water molecule was lost with further heating. Since the carbonyl group of anhydrides was more electrophilic than the acidic group in a nucleophilic addition reaction, the interaction with the -OH of dextrin via esterification was favored. Therefore, the grafting of the citrate group occurred in the cyclodextrin (CD) chain, and monoesters were formed (Figure S1 b)). It formed a new carboxylic acid unit in the citric acid that could form a new intra-molecular anhydride moiety with the adjacent carboxylic acid unit (Figure S1 c)). Therefore, the grafted functional group may react with the -

OH of another dextrin chain, resulting in cross-linking step (Figure S1 d)). The esterification of CDs with citric acid was accelerated by sodium hypophosphite monohydrate as a catalyst and took place at temperatures lower than 140 ⁰C. Based on the proposals of earlier studies [79–81], the current research developed the synthesis with curing temperatures lower than 140 ⁰C to confirm the proposed cross-linking mechanism (Figure S1). The cross-linking process occurred notwithstanding the temperature value, and it became faster when the system was more anhydrous.

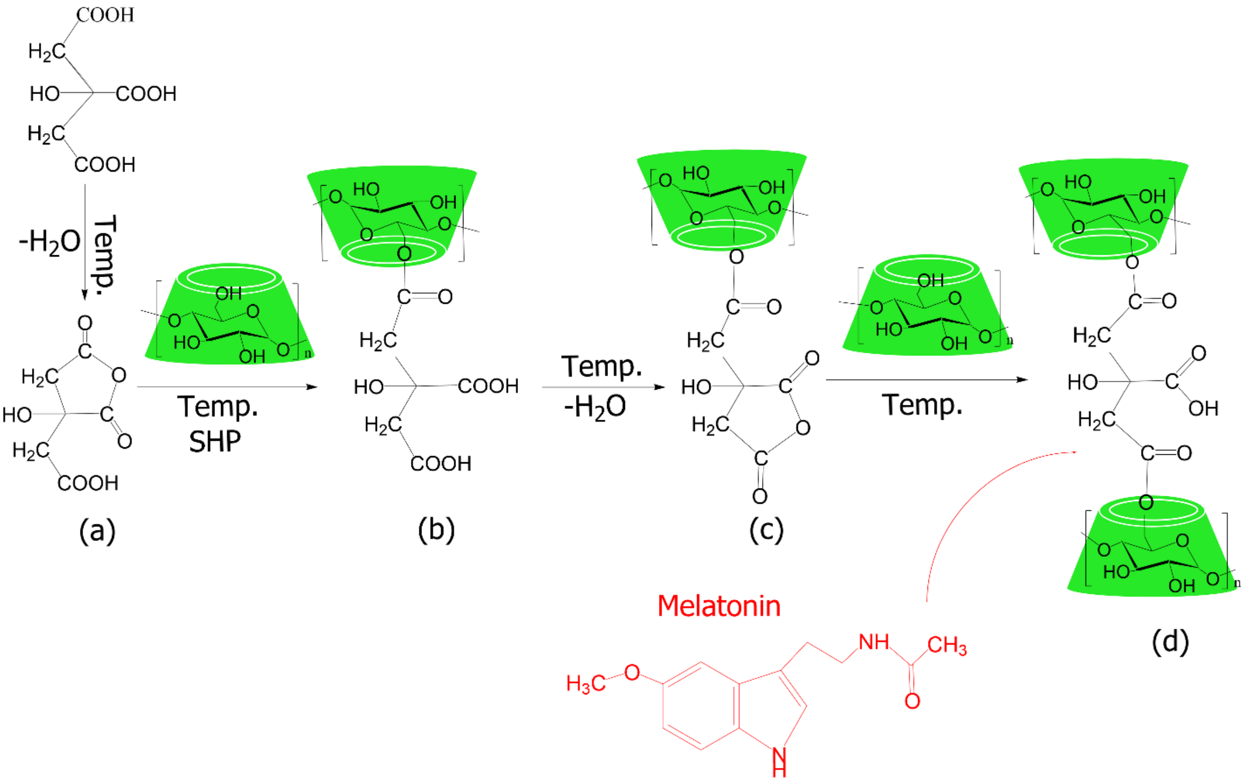

Figure S1. Formation of cyclodextrin-based molecularly imprinted and non-molecularly imprinted nanosponges (CD-MIPs-based NSs and CD-NIPs-based NSs). Mechanism of the cross-linking of dextrin by CA: (**a**) creation of cyclic intermediate (≤ 140 ⁰C), (**b**) reaction of dextrin with the intermediate in the presence of SHP (≤ 140 ⁰C), (**c**) formation of a new intra-molecular anhydride moiety with the adjacent carboxylic acid unit, and (**d**) cross-linking of dextrin by CA (≤ 140 ⁰C).

S3. Melatonin stability

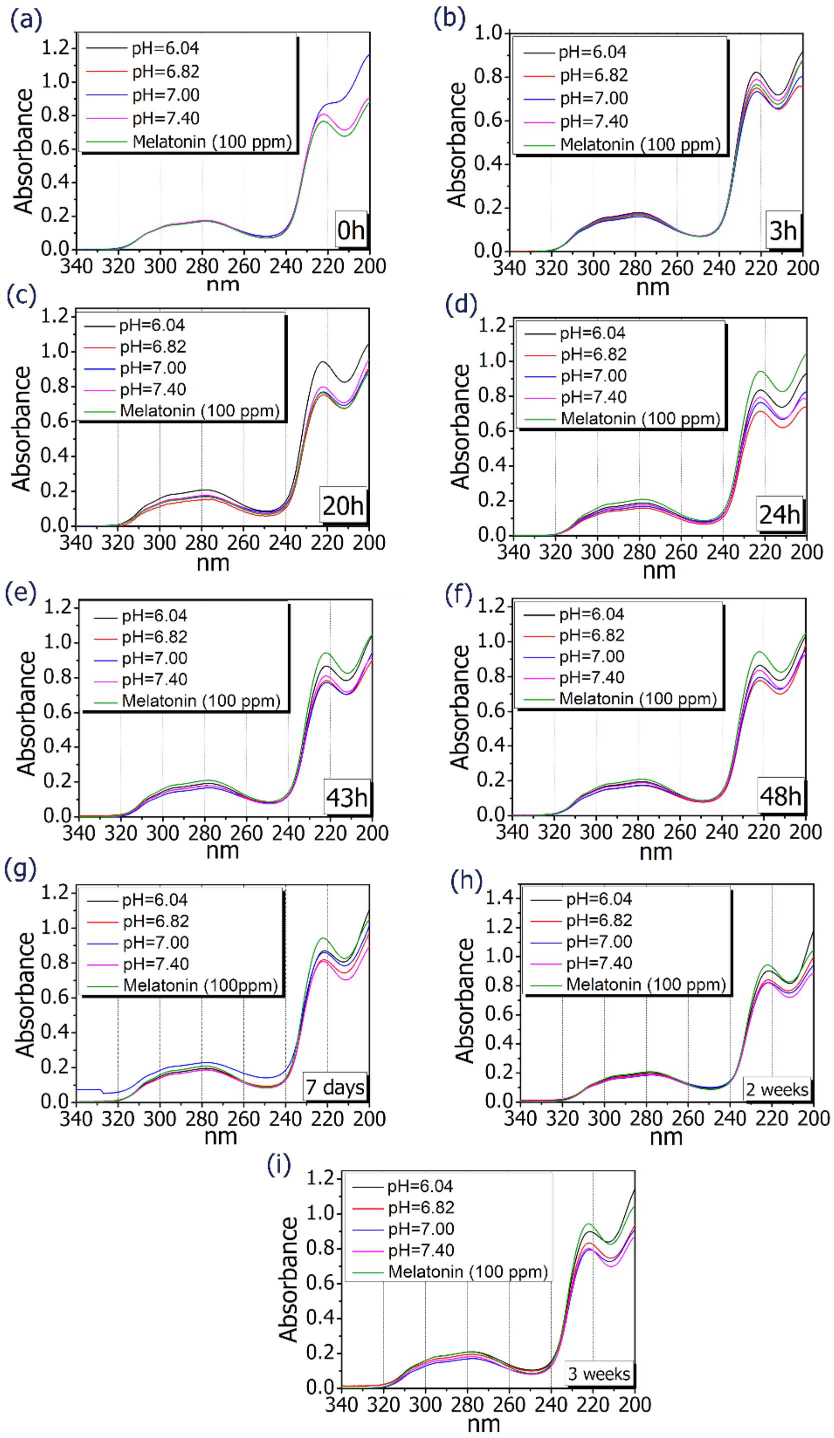

Figure S2. UV-VIS spectra of melatonin aqueous solution (100 ppm); melatonin solution at pH 6.0, melatonin solution at pH 6.8, melatonin solution at pH 7, melatonin solution at pH 7.4, after (**a**) 0h, (**b**) 3h, (**c**) 20h, (**d**) 24 h, (**e**) 43h, (**f**) 48h, (**g**) 7 days, (**h**) 2 weeks, and (**i**) 3 weeks.

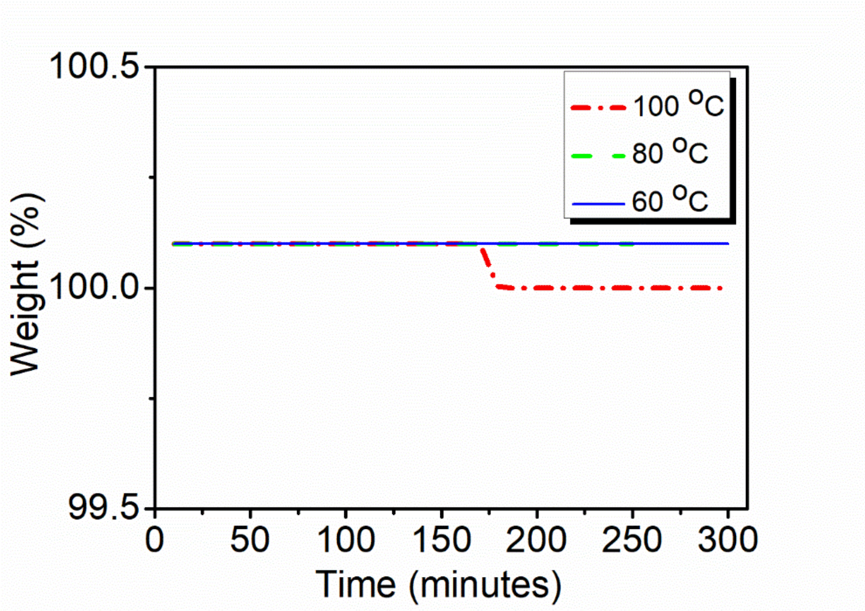

Figure S3. Isothermal TGA curve of melatonin during heating at 100 ⁰C, 80 ⁰C, and 60 ⁰C for 5 hours (300 minutes).

S4. Thermogravimetric analysis (TGA)

The first percentage of mass loss was related to the loss of water associated with polymer, observed at temperatures up to 100 ⁰C, and being 4.26% of NIP βCD: CA_1: 8, 5.25% of MIP βCD: CA_1: 8_20% Melatonin, and 5.83% of MIP βCD: CA_1: 8_50% Melatonin, as presented in Figure 4 a). The maximum degradation processes of the β-CD, 83.82% occurred at around 256 to 390 ⁰C. The thermogram of NIP βCD: CA_1: 8 presented two degradation steps. The first one, being 15.95% from 158 to 220 ⁰C, could be related to the degradation of the drugs. The second and main weight loss step, 40.31% from 220 to 415 ⁰C, was related to the degradation of the NSs. The thermogram of MIP βCD: CA_1: 8_20% Melatonin presented two degradation steps. The first one of 13.92% presented from 133 to 220 ⁰C. The second and main weight loss step, 47.15%, presented from 220 to 484 ⁰C. Furthermore, the first degradation step of the thermogram of MIP βCD: CA_1: 8_50% Melatonin, being 12.36%, appeared from 139 to 220 ⁰C. Whereas the second and main weight loss step, 42.19%, presented from 220 to 423 ⁰C.

The maximum degradation processes of the M-βCD, 88.40%, occurred at around 278 to 372 ⁰C. The TGA results in Figure 4b show that the first weight-loss, being 3.81% of NIP M-βCD: CA_1: 8, 4.52% of MIP M-βCD: CA_1: 8_20% Melatonin, and 4.73% of M-βCD: CA_1: 8_50% Melatonin at 100 ⁰C, corresponded to the evaporation of water associated with the polymer. Similarly, as the thermograms of βCD-based MIP-NSs or βCD-based NIP-NSs, M-βCD-based MIP-NSs or M-βCD-based NIP-NSs presented two degradation steps. NIP M-βCD: CA_1: 8 presented the first one of 11.71% from 118-215 ⁰C; MIP M-βCD: CA_1: 8_20% Melatonin presented the first one of 15.64% from 113-200 ⁰C; and MIP M-βCD: CA_1: 8_50% Melatonin presented the first one of 15.02% from 127-205 ⁰C. The weight loss related to the maximum degradation, 44.55% of NIP M-βCD: CA_1: 8, occurred between 214 ⁰C and 407 ⁰C; 47.04% of MIP M-βCD: CA_1: 8_20%

Melatonin occurred between 200 ⁰C and 422 ⁰C; and 47.36% of MIP M-βCD: CA_1: 8_50% Melatonin occurred between 208 ⁰C and 429 ⁰C.

S5. Differential Scanning Calorimetry (DSC)

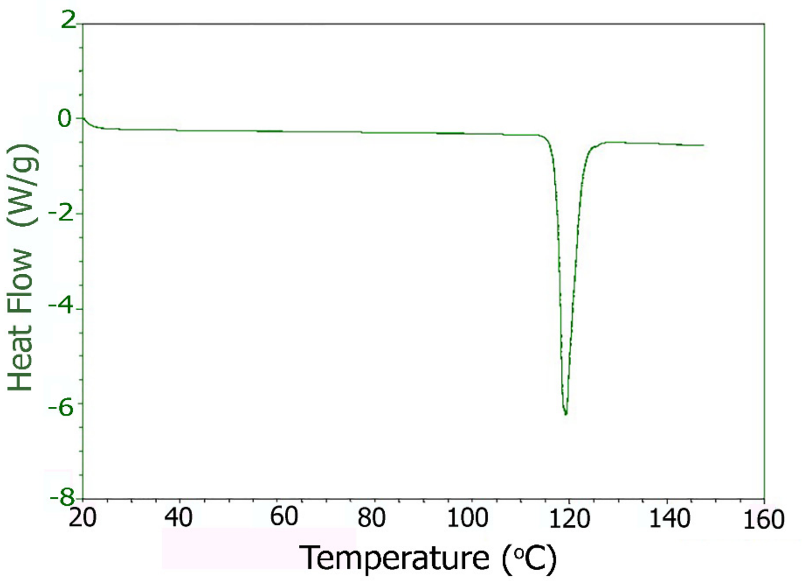

Figure S4. Differential scanning calorimetry (DSC) thermograms of melatonin.

S6. Fourier Transform Infrared Spectroscopy (FTIR) Analysis

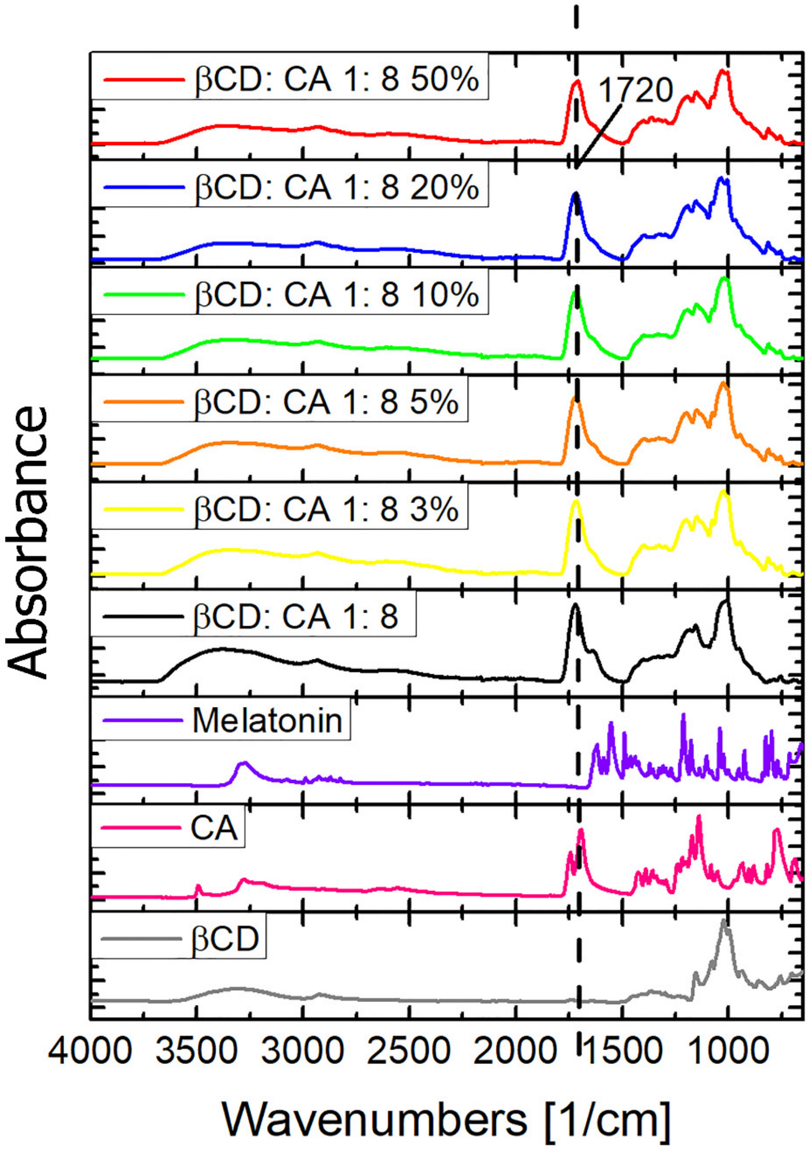

Figure S5. FTIR spectra of β-Cyclodextrin (β-CD)-based MIP-NSs and NIP-NSs (Melatonin 3%, 5%, 10%, 20%, and 50%), Melatonin, CA, and β-CD.

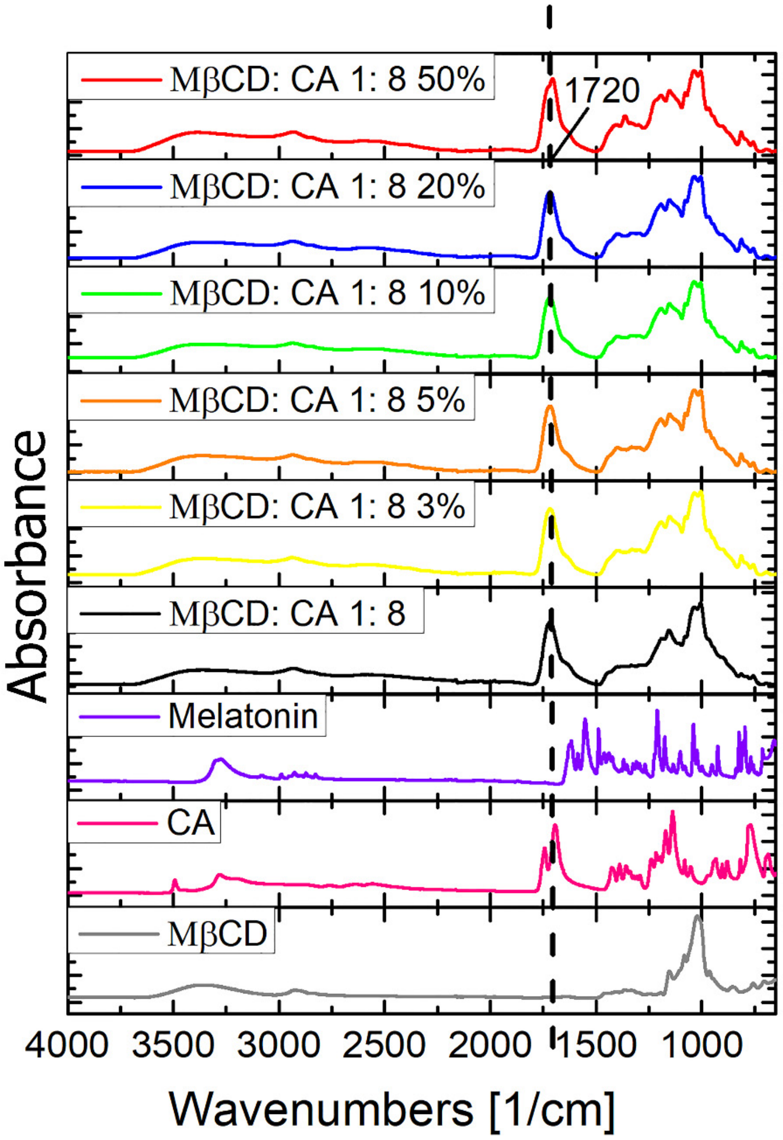

Figure S6. FTIR spectra of M-βCyclodextrin (M-βCD)-based MIP-NSs and NIP-NSs (Melatonin 3%, 5%, 10%, 20%, and 50%), Melatonin, CA, and M-βCD.

S7. Evaluation of the pharmaceutical parameters of cream formulations

Table S1. The spreadability and the viscosity of the cream formulations.

Cream Formulations	Spreadability (g*cm)/s	Viscosity (Pa.s)
Cream	0.11	3.24
MIP β-CD:CA_1:8/10% Melatonin + Cream	0.13	17.75
MIP β-CD:CA_1:8/50% Melatonin + Cream	0.29	10.82
MIP M-βCD:CA_1:8/10% Melatonin + Cream	0.06	52.14
MIP M-βCD:CA_1:8/50% Melatonin + Cream	0.06	8.54

Table S2. The pH of the cream formulations.

Cream Formulations	pH
Cream	4.90
MIP β-CD:CA_1:8/10% Melatonin + Cream	3.38
MIP β-CD:CA_1:8/20% Melatonin + Cream	3.58
MIP β-CD:CA_1:8/50% Melatonin + Cream	3.95
MIP M-βCD:CA_1:8/10% Melatonin + Cream	3.05
MIP M-βCD:CA_1:8/20% Melatonin + Cream	3.42
MIP M-βCD:CA_1:8/50% Melatonin + Cream	3.91

S8. The release study *in vitro*

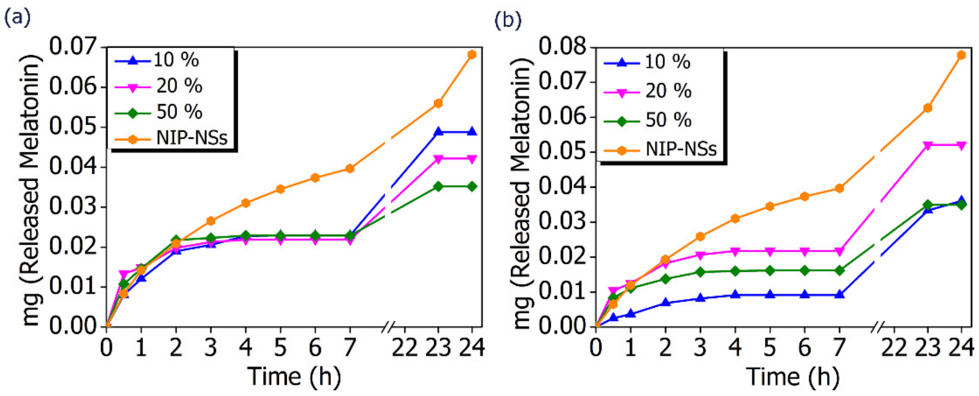

Figure S7. *In vitro* release curves of melatonin loaded into MIP-NSs (10%, 20%, and 50% Melatonin), and NIP-NSs (20% Melatonin). The amount of melatonin (mg) released from 100 mg of NS over time. (**a**) βCD-based NIP-NSs and βCD-based MIP-NSs; and (**b**) M-βCD-based NIP-NSs and M-βCD-based MIP-NSs.
